# Detection of G-quadruplex DNA in mammalian cells

**DOI:** 10.1093/nar/gkt957

**Published:** 2013-10-24

**Authors:** Alexander Henderson, Yuliang Wu, Yu Chuan Huang, Elizabeth A. Chavez, Jesse Platt, F. Brad Johnson, Robert M. Brosh, Dipankar Sen, Peter M. Lansdorp

**Affiliations:** ^1^Terry Fox Laboratory, British Columbia Cancer Agency, Vancouver, BC V5Z 1L3, Canada, ^2^Laboratory of Molecular Gerontology, National Institute on Aging, National Institutes of Health, NIH Biomedical Research Center, Baltimore, MD 21224, USA, ^3^Department of Chemistry, Simon Fraser University, Burnaby, BC V5A 1S6, Canada, ^4^Department of Pathology and Laboratory Medicine, University of Pennsylvania, Philadelphia, PA 19104-6100, USA, ^5^Division of Hematology, Department of Medicine, University of British Columbia, Vancouver, BC V6T 1Z4, Canada and ^6^European Research Institute for the Biology of Ageing, University of Groningen, University Medical Centre Groningen, A. Deusinglaan 1, NL-9713 AV Groningen, The Netherlands

## Abstract

It has been proposed that guanine-rich DNA forms four-stranded structures *in vivo* called G-quadruplexes or G4 DNA. G4 DNA has been implicated in several biological processes, but tools to study G4 DNA structures in cells are limited. Here we report the development of novel murine monoclonal antibodies specific for different G4 DNA structures. We show that one of these antibodies designated 1H6 exhibits strong nuclear staining in most human and murine cells. Staining intensity increased on treatment of cells with agents that stabilize G4 DNA and, strikingly, cells deficient in FANCJ, a G4 DNA-specific helicase, showed stronger nuclear staining than controls. Our data strongly support the existence of G4 DNA structures in mammalian cells and indicate that the abundance of such structures is increased in the absence of FANCJ. We conclude that monoclonal antibody 1H6 is a valuable tool for further studies on the role of G4 DNA in cell and molecular biology.

## INTRODUCTION

Single-stranded guanine (G)-rich DNA can form stable secondary structures *in vitro* called G-quadruplex (G4) DNA (1,2). G4 DNA is generated through the association of four guanines bound through Hoogsteen base pairing and characterized by variable stacks of guanine quartet planes, strand orientation, glycosidic bond angles and stabilizing cations (3). Putative G4-forming sequences are proposed to form functionally relevant G4 DNA structures throughout the genome including immunoglobulin switch regions, promoter sequences, rDNA and telomeric repeats (4,5). However, in theory, G4 DNA can arise anywhere in the genome where sufficiently long stretches of single-stranded G-rich DNA are exposed during replication, transcription or recombination (6). Detailed *in vitro* chemical analysis of quadruplex-forming oligonucleotides has revealed the existence of a plethora of dynamic quadruplex structures with varying stabilities (3,7–12). The structural polymorphism of G4 DNA could make these structures valuable molecular targets to study biological processes and for possible therapeutic intervention (3).

Interest in G4 DNA has been increased by the discovery that stabilized quadruplex structures negatively affect enzyme-catalyzed elongation of telomeric sequences *in vitro* (13). Given that up to 90% of all cancers rely on the activity of telomerase for continued growth, control of telomerase-mediated telomere elongation through G4 DNA stabilization is perceived as having therapeutic potential. The potential to inhibit telomerase for cancer therapy has spurred the development of small molecules that target and stabilize G4 DNA. Treatment of various cancer cell lines with such ligands was found to result in telomere shortening and senescence, supporting that stabilization of G4 DNA structures can perturb telomere homeostasis and potentially suppress tumor growth (14). Moreover, a number of human genetic diseases are characterized by telomere defects, and it has been proposed that G-quadruplex structures forming either at the 3′ end of telomeres or during telomere replication play a role in such diseases (15,16). Despite these postulated connections between G4 DNA and human disease, there is to date limited direct evidence for the existence of G4 DNA in human cells.

Here we report the development and characterization of novel monoclonal antibodies specific for distinct structural variants of G4 DNA. Immunofluorescence microscopy studies using one of these, designated 1H6, showed nuclear staining in most human cells, which was suppressed by the addition of soluble G4 DNA and abolished with prior treatment with DNase. Treatment of cells with G-quadruplex stabilizing small molecules 5,10,15,20-tetra(*N*-methyl-4-pyridyl)–porphyrin (TMPyP4) or telomestatin (TMS) increased the number and intensity of the nuclear staining by 1H6. Furthermore, vertebrate cells deficient for the G4 resolving helicase FANCJ that were exposed to TMS showed stronger 1H6 nuclear staining than controls, supporting the notion that G4 DNA is enriched as a consequence of a genetic deficiency.

Taken together, our results support the presence of abundant G4 DNA in most mammalian cells. We propose that 1H6 and the other monoclonal antibodies against G4 structures described in this report are valuable analytical tools for further studies on the biological and therapeutic role of G4 DNA *in vivo*.

## MATERIALS AND METHODS

### Polyacrylamide gel analysis and circular dichroism studies

G4 DNA secondary structure was assessed by native gel electrophoresis with a 12–15% polyacrylamide gel containing 10 mM NaCl or 10 mM K_2_B_4_O_7_. Samples were visualized by UV shadow or Gel Red (Biotum) staining. Bands corresponding to both G4 DNA and unstructured monomers were excised. Each excised gel fragment was crushed into 25 ml of elution buffer (50 mM Tris–HCl (pH 7.5), 1 mM EDTA, 25 mM NaCl) and was shaken overnight at 4°C. Eluted DNA was syringe-filtered from gel pieces and precipitated by repeated rounds of 2-butanol extraction and standard ethanol precipitation. Purified G4 samples were resuspended in phosphate-buffered saline (PBS) and monomers were resuspended in TE. Both were stored at −20°C. Samples prepared for circular dichroism (CD) analysis were diluted in PBS or TE to a final concentration of 5–10 μM (70–95 μg/ml). CD spectra were measured using a Jasco-810 spectropolarimeter at 25°C. Readings were recorded over a wavelength range of 200–320 nm in a quartz cuvette with a 1-cm path length. Measurements were averaged between three accumulations with an instrument scanning speed of 200 nm/min and readings were blank-corrected with buffer alone.

### Specificity

Antibody specificity was screened by a direct ELISA assay where biotinylated G4 structures were captured by SA-coated plates while double-stranded DNA, single-stranded DNA and various proteins were coated to the plates. Purified antibodies were subsequently added at a half maximal binding concentration and incubated on pre-coated plates for 1 h at room temperature. Bound antibodies were detected using goat anti-mouse HRP and revealed with the ABTS substrate and were quantified by spectrophotometry at 405 nm. Antibody 1H6 underwent another round of specificity testing by competition ELISA. The relative binding specificity by the 1H6 antibody was determined in triplicate measurements by pre-incubation with different competitors at room temperature for 1 h. The 1H6 antibody concentration was below half maximal binding concentration and constant in each competitive reaction. All competing agents were 1000-fold molar excess over 1H6 antibody. Plates coated with immobilized G4-DNA were used to evaluate percentage competition in comparison with incubated antibodies without competitor. A minimum of 4 μg of synthetic DNA or protein was loaded per competition. All competition reactions were incubated in PBS containing 1% BSA and monomeric samples were initially denatured at 95°C for 2 min to maintain single-stranded form. Anti-DNA antibodies were detected with HRP labeled goat anti-mouse IgG and revealed with ABTS substrate. The percentage of competition = [(OD control−OD with competitor)/OD control] × 100.

### Immunofluorescence microscopy

Cells were grown on flamed coverslips in 12-well plates and incubated at 37°C. For some experiments, cells were incubated with TMPyP4 (Calbiochem) or TMS (17) for 24 h before fixation. For proteinase K digestion, cells were pretreated with 10 mg/ml proteinase K for 1 h at 37°C. Cells were fixed in 4% paraformaldehyde (PFA, Alfa Aesar) in PBS and then permeabilized with 0.5% Tween 20 in PBS. Cells were treated with 20 µg/500 µl RNase A (Invitrogen) and subsequently blocked in image-iTFX (Invitrogen) and 5% normal goat serum (Sigma) for 12 h at 4°C. Cells were then incubated with purified G4 antibodies either overnight at 4°C or 2 h at room temperature. Cells were washed with PBST and then incubated with either: Phycoerythrin goat anti-mouse IgG (Jackson Immuno Research), Cy3 donkey anti-mouse IgG (Jackson Immuno Research), Alexa Fluor 596 goat anti-mouse IgG (Invitrogen) followed by staining for DNA with 20 ng/ml Dapi (Sigma). Coverslips were washed, mounted in Vectashield (Vector Laboratories, Inc.) or Prolong gold antifade (Invitrogen). For DNase experiments; BJ-hTERT cells were grown on slides, fixed in 4% PFA in PBS for 10 min at room temperature, permeabilized in 0.2% Triton X-100 in PBS for 1 min and washed in PBS. They were then incubated for 2 h at 37^°^C in 40 mM Tris Cl (pH 8), 5 mM CaCl2, 2 mM MgCl2, 100 ug/ml BSA alone or including 0.06 U/ul of DNase I (RQ1 DNase, Promega) and 80 gel units/microlitre of micrococcal nuclease, washed in PBS and then stained with antibodies. Rabbit anti-fibrillarin was from Abcam (Ab5821) and was used at 1:100. For the confocal microscopy, the exposures were identical for the control and nuclease-treated samples. Final image brightness was adjusted in Photoshop identically for the control and nuclease-treated samples.

## RESULTS AND DISCUSSION

### Generation of distinct quadruplex structures

**Figure 1.**
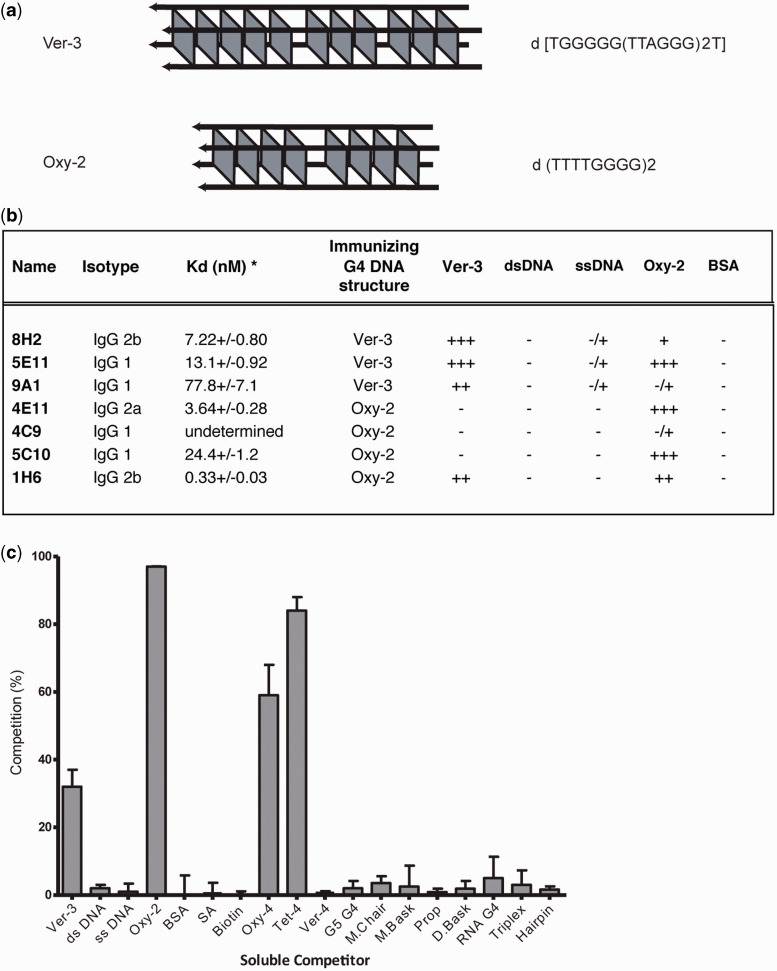
Immunizing antigens and antibody characteristics. (**a**) Two different tetramolecular G4 DNA structures were generated for the purposes of immunizing animals:er-3 [TGGGGG(TTAGGG)_2_T] and Oxy-2 (TTTTGGGG)_2_. (**b**) The majority of purified monoclonal antibodies that bind G4 DNA *in vitro* are IgG1 and have low nanomolar apparent affinities by ELISA. Purified antibodies bind with high affinity to tetramolecular G4 DNA structures and have limited binding to single-stranded or double-stranded DNA. Single-stranded (ssDNA) and double-stranded (dsDNA) DNA in these experiments were ssDNA oligo’s (used for preparing G4 DNA for immunization) before and after annealing to their complementary sequence. *Kd measurement of binding to immunizing G4 structure and Kd standard deviation based on triplicate measurements by ELISA. OD cutoffs <0.1, 0.1–0.25, 0.25–0.5, 0.5–0.75, >0.75 (−, −/+, +, ++, +++). (**c**) The1H6 antibody binds multiple G-quadruplex structures. Specificity testing by competition ELISA of monoclonal antibody 1H6 characterized by promiscuous binding to varying soluble competitors. Competitor sequences and structures are listed in Supplementary Table S1. The 1H6 antibody binds to tetramolecular structures and unimolecular structures without sequence specificity. Error bars represent the standard deviation of triplicate experiments. Soluble competitors that compete for binding the 1H6 antibody include tetramolecular G4 DNA (Ver-3 and Oxy-2) and unimolecular G4 DNA (Oxy-4 and Tet-4) also shown in Supplementary Table S1. Single-stranded (TTTTGGGG)_2_ and its complement were used to test 1H6 specificity binding to ssDNA and dsDNA.

Telomeric DNA typically comprises G-rich repeats (18). In addition, the 3′ distal portion of most telomeres is naturally single-stranded and could form G4 structures *in vivo* (19–24). Therefore, we chose to generate stable G-quadruplex structures from oligonucleotides containing vertebrate telomeric repeats (TTAGGG) or ciliate telomeric repeats (*Oxytricha,* GGGGTTTT, Figure 1A). G4 structures were separated from monomeric DNA using native polyacrylamide gel electrophoresis (2). All sequences used to generate G4 structures are listed in Supplementary Table S1.

To differentiate between higher-order nucleic acid structures that are not readily resolved by native polyacrylamide gel electrophoresis alone, we characterized all purified nucleic acid structures by CD spectropolarimetry. We compared the patterns of our purified G4 structures with known reference spectra of specific well-defined G4 structures (25–27). Both *Oxytricha* (Oxy-2) and vertebrate (Ver-3) sequences folded into characteristic parallel G4 DNA structures, with ellipticity maxima and minima at ∼265 and 240 nm, respectively (Supplementary Figure S1).

### High affinity monoclonal antibodies recognize specific G4 DNA structures

Spleen cells from mice immunized with stable G4 DNA structures were hybridized with murine Sp2/OAg14 myeloma cells to obtain hybridomas secreting monoclonal antibodies. Several clones were identified by screening supernatants in ELISA assays. Following subcloning, several stable monoclonal antibody secreting hybridomas were obtained (Figure 1B). The avidity of the purified antibodies was tested in titration experiment with their respective immunogen (Supplementary Figure S2). Most antibodies were determined to bind antigen in the low nanomolar range (0.3–78 nM, Figure 1B). Monoclonal antibodies raised against the tetramolecular Ver-3 G4 DNA structure (designated 8H2 and 9A1) demonstrated high specificity for the Ver-3 G4 DNA structure (Figure 1B). Interestingly, 5E11 displayed significant binding to the Oxy-2 tetramolecular structure. None of the Ver-3 specific antibodies 5E11, 8H2 or 9A1 significantly bound double-stranded or single-stranded DNA in our assay (Figure 1B). Antibodies 4E11 and 5C10 raised against tetramolecular Oxy-2 G4 DNA, demonstrated high selectivity for the tetramolecular Oxy-2 G4 DNA structure (Figure 1B), whereas 1H6 bound both tetramolecular structures generated from Oxy-2 or Ver-3 sequences (Figure 1B). None of the antibodies raised against tetramolecular Oxy-2 G4 DNA significantly bound double-stranded or single-stranded DNA, supporting that these antibodies distinguish between different G4 DNA structures.

To further characterize the antibodies that bound multiple tetramolecular topologies (5E11 and 1H6), we performed competition ELISA assays with an increased pool of G4 DNA structures. Although the 5E11 antibody bound all tetramolecular structures, it did not bind any unimolecular or bimolecular structures tested (data not shown). Interestingly, the 1H6 antibody that had been previously shown to bind to both tetramolecular structures from Oxy-2 and Ver-3 also bound to the unimolecular structures from Oxy-4 and Tet-4 (Figure 1C). However, the 1H6 antibody did not seem to bind the unimolecular Ver-4 structure. These binding properties suggest that the 1H6 antibody has affinity for some, but not all, G4 DNA structures. By contrast, the 1H6 antibody did not significantly bind a tetramolecular RNA structure or a triplex DNA structure and displayed minimal binding to either single-stranded or double-stranded DNA (Figure 1C). These results support that 1H6 antibody has broad specificity for many, but not all, G-quadruplex structures tested.

To aid in future studies and elucidate the binding characteristics of the monoclonal antibodies for these G4 DNA structures, we deduced the amino acid sequence for the variable heavy (V_H_) and variable light (V_L_) chain of the hybridomas from the corresponding genes (Supplementary Table S2). Sequence data are available online from GenBank, accession numbers; KC414123-KC414132.

### Monoclonal antibody 1H6 exhibits nuclear staining that is sensitive to treatment with DNase

**Figure 2.**
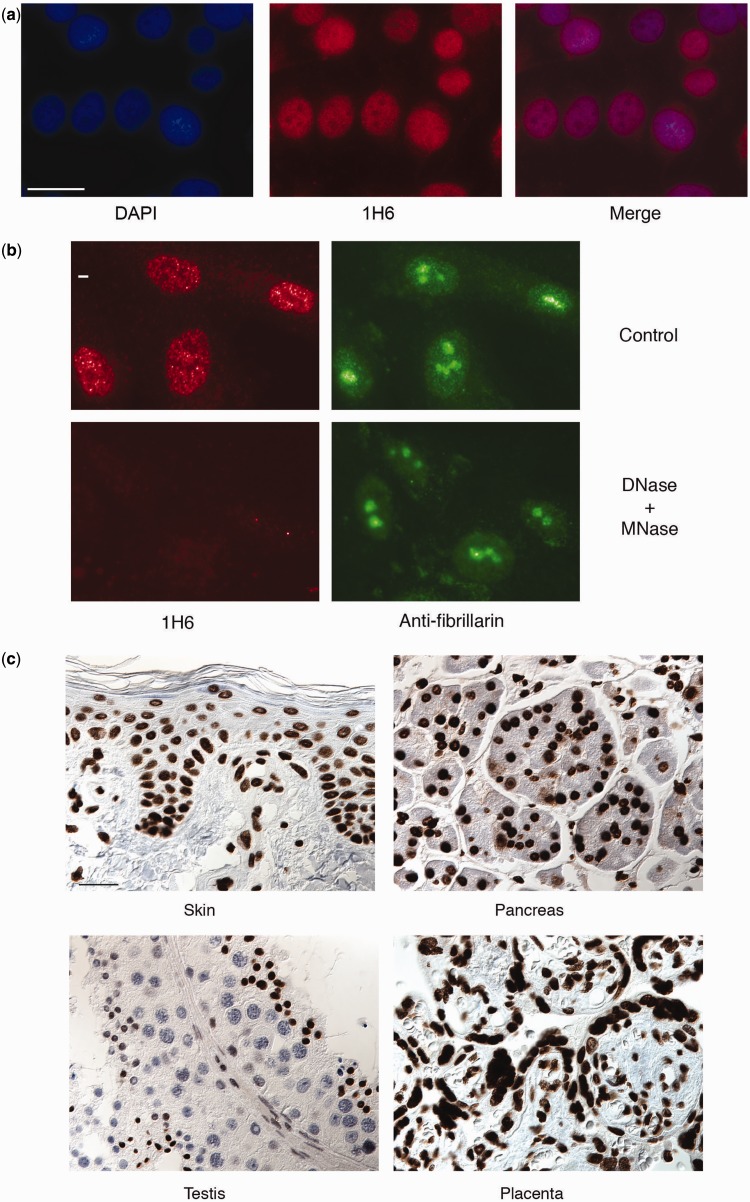
The 1H6 antibody generates discrete nuclear foci in human cell lines, which is sensitive to treatment with DNase. (**a**) Representative images of immunofluorescence analysis using the 1H6 antibody in human cells (HeLa). Bar represents 50 μm. (**b**) Fluorescent microscopy analysis of HeLa cells stained with 1H6 (red), DAPI DNA dye (Blue) and anti-fibrillarin (green) before and after treatment of cells with DNase. Digestion with RQ1 DNase and micrococcal nuclease show loss of 1H6 foci. Control experiments stained with rabbit anti-fibrillarin do not show loss of signal on digestion with RQ1 DNase and micrococcal nuclease. Bar represents 5 μm. (**c**) Normal human tissue sections were used for immunohistochemical staining with 1H6 antibodies (dark brown). Representative images from the skin, pancreas, testis and placenta are shown (other tissues can be see in Supplementary Figure S3 online). Bar represents 50 μm.

Previously, several antibodies and antibody fragments to various G4 DNA structures have been generated (28–31). Single-chain phage display antibodies demonstrated replication-dependent resolution and formation of G4 DNA at the abundant (<10^6^) telomeres of the cilated protozoa *Stylonychia* (30) and, more recently, nuclear staining of mammalian cells with a different phage display antibody was reported (31). To determine if any of our antibodies could recognize G4 DNA *in vivo*, we tested our antibodies in various vertebrate cell lines using fluorescence microscopy. The 1H6 antibody exhibited granular nuclear staining on HeLa cells (Figure 2A). Some variability in the intensity of the nuclear staining was observed between cells. The strongest staining was observed in cells on slides that were permeabilized before fixation, indicating that accessibility to antigen is probably limiting 1H6 nuclear staining. Granular 1H6 staining was observed in nuclei of cells that do not express telomerase (BJ primary fibroblasts, not shown), telomerase-positive cells (HeLa, Figure 2A) and in telomerase-independent (alternative lengthening of telomeres, ALT) GM847 cells (not shown) indicating that the antigen being detected *in vivo* by 1H6 is neither cancer specific nor telomerase dependent. To further study the nature of the antigen recognized by 1H6 slides with fixed cells were subjected to treatment with various enzymes. Neither proteinase K nor RNase treatment had noticeable effect on 1H6 staining (not shown), whereas treatment with DNase completely abolished 1H6 staining but not the staining with anti-fibrillarin used as a control (Figure 2B). Further studies of 1H6 of cells in various human tissues also showed strong nuclear staining of most cells (Figure 2C and Supplementary Figure S3).

A noticeable exception was the testis where some cells appeared to show less or no staining (Figure 2C, bottom left panel). Cells fixed with methanol or with methanol and acetic acid also showed nuclear staining, indicating that the 1H6 antigen is resistant to organic solvents (results not shown). Collectively, the data from experiments with different fixation techniques and enzyme digestions suggest that the granular nuclear staining detected by 1H6 is primarily of DNA origin.

### Staining of chromosomes and DNA fibers by 1H6

**Figure 3.**
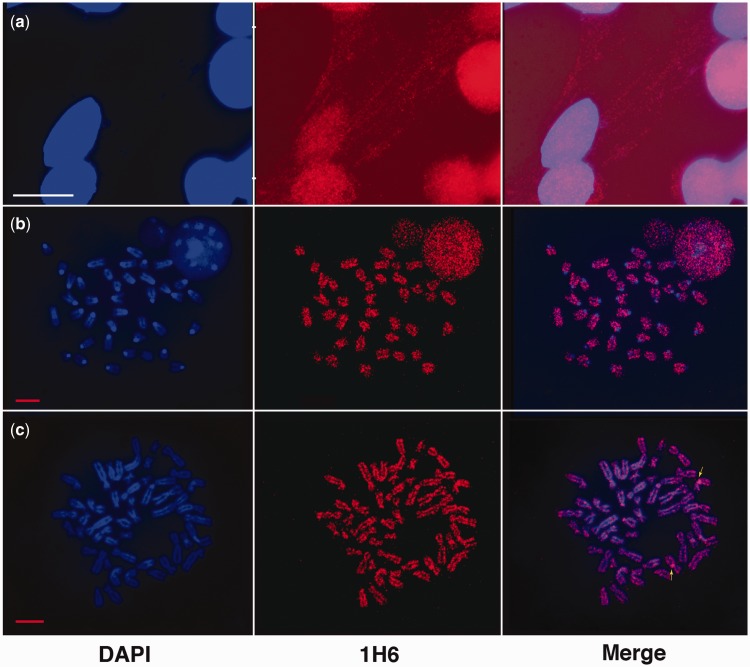
Punctate fine granular staining of chromosomes and DNA fibers with 1H6. (**a**) Representative images of paraformaldehyde-fixed HeLa cells previously lyzed with Triton X-100. The1H6 antibody (red) appears to be located on nuclear DNA and DNA fibers (blue fibers stained with DAPI) emanating from the nucleus. Image has DAPI channel amplified to identify DNA fibers more clearly. Bar represents 50 µm. (**b**) The1H6 antibody shows many discrete foci on metaphase chromosomes of normal mouse emrbryonic stem cells. (**c**) The 1H6 antibody shows many discrete foci on chromosomes of human metaphase preparations. Arrows point to bright staining near the centromere of chromosome 9. Representative images of immunofluorescence analysis of metaphase preparations using 1H6 antibodies. Bar represents 10 μm.

To further explore the 1H6 staining within the nucleus, we sought to explore the staining of chromosomes and DNA fibers. Strikingly, when cells were lysed with Triton X-100 before formaldehyde fixation, streaks of fine granular 1H6 staining were observed spooling out of the nucleus (Figure 3A). Our interpretation of this observation is that following Triton-X lysis, 1H6 binding sites that are otherwise masked become accessible. Metaphase preparations of normal mouse ES cells (Figure 3B) and human HeLa cells (Figure 3C) also showed an irregular fine granular staining pattern. Based on our observation with DNA fibers (Figure 3A), we assume that only a fraction of 1H6 sites is detected in the highly compacted DNA of metaphase chromosomes. In general, the number of fine granular nuclear foci greatly exceeded the number of telomeres in cells (Figure 3B and C). Our observations support that 1H6 antigens are located throughout the genome and not exclusively at telomeres in accordance with bioinformatic studies examining the potential location of G4 DNA across the human genome (4,5,19). Our finding that metaphase chromosomes as well as most interphase cells are labeled by the 1H6 antibody furthermore suggests that the G4 DNA structures recognized by this antibody are not transiently formed during replication, transcription or recombination but are more stably present at selected genomic regions. Unfortunately our attempts to confirm specificity of 1H6 for G-rich DNA and define the genomic sites capable of forming G4 DNA using chromatin immunoprecipitation (32) have so far been unsuccessful. Whether loss of antibody binding on isolation or size fractionation of DNA or failure to amplify and sequence G4 DNA structures is responsible for our lack of success is currently not known.

### The 1H6 staining pattern is modulated by synthetic G4 structures and G4 stabilizing agents

**Figure 4.**
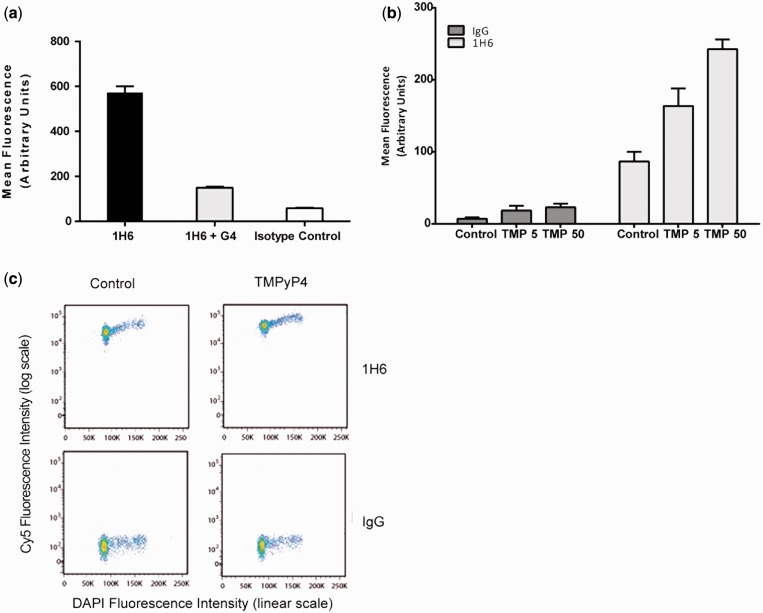
The 1H6 foci decrease in the presence of G4 DNA and increase in the presence of TMP and TMS. (**a**) Synthetic tetramolecular G4 DNA (Oxy-2 tetraplex) decreases 1H6 nuclear foci as detected by flow cytometry. Human HeLa cells were prepared and stained with anti-G-quadruplex antibody 1H6 with and without the presence of synthetic tetramolecular G4 DNA. Mean fluorescence was measured by flow cytometry and plotted above. Incubating the 1H6 antibody in the presence of tetramolecular DNA decreases the total mean fluorescence (n > 25 000). (**b**) Flow cytometry quantitative analysis of HeLa cells show increased mean fluorescence on treatment with TMPyP4. HeLa cells treated with increasing concentrations of TMPyP4 and subsequently stained with the 1H6 antibody. Increased TMPyP4 treatment resulted in increased mean fluorescence detected for 1H6 but not controls. TMPyP4 concentrations (TMP) in µM are listed below (n > 25 000). (**c**) Flow cytometry analysis of HeLa cells treated with TMPyP4 or no drug control. Log scale differences observed on treatment in cells stained with 1H6 as compared with controls (n > 20 000).

To further investigate the nuclear staining observed with 1H6 antibody, we used soluble, synthetic G4 DNA in competition experiments. At equimolar concentrations, synthetic G4 DNA significantly reduced the nuclear fluorescence, an observation that was confirmed and quantified by flow cytometry (Figure 4A). Increasing concentrations of synthetic G4 DNA did not change this pattern. The competition between *in vivo* DNA and synthetic G4 DNA provides further support for the notion that the 1H6 antibody detects G4 DNA structures in human cells.

It has been postulated that G4 DNA can form throughout the genome and that these structures must be resolved to preserve genomic integrity throughout multiple rounds of DNA replication and transcription (33). Unresolved G4 DNA could potentially stall replication forks (34) or interfere with gene expression (35). We hypothesized that treatment of cells with small molecules that stabilize G4 DNA would increase the number of nuclear foci detected by 1H6. Therefore, we treated HeLa cells with increasing concentrations of TMPyP4 [Mesotetra(N-methyl-4-pyridyl)porphine], a compound suggested to bind and stabilize G4 DNA. The mean fluorescence of the cells was measured by flow cytometry (Figure 4B and C). Consistent with immunofluorescence experiments, we detected an increased mean fluorescence signal with increasing amounts of TMPyP4. Of note, nuclei at all stages of the cell cycle showed increased fluorescence (Figure 4C). In addition, time-course experiments with the human cell line U2OS treated with TMS (17), another G4 stabilizing compound, showed increased number of antibody foci over time (Supplementary Figure S4A and B). Taken together, these experiments suggest that agents that increase G4 DNA stability increase staining by 1H6 and that recognition of G4 structures by this antibody is not affected by the G4 binding ligands tested.

### FANCJ deficiency results in increased nuclear staining by 1H6

The adverse effects of various G quadruplex structures formed during replication and recombination are presumed to be countered by specialized helicases. Interestingly, a number of helicase proteins have been described to specifically catalyze the ATP-dependent unwinding of G4 DNA structures (15) (36). RecQ helicase family members WRN, BLM and Sgs1 have all been shown to unwind numerous G4 DNA structures *in vitro* (37–39), and deficiencies in these helicases cause changes in gene expression preferentially at loci containing sequences capable of forming G-quadruplexes (40,41). In addition to the 3′–5′ helicases of the RecQ family there are also 5′–3′ helicases, including human FANCJ, Pif1 and DNA2 that can unwind G4 DNA (15,35).

**Figure 5.**
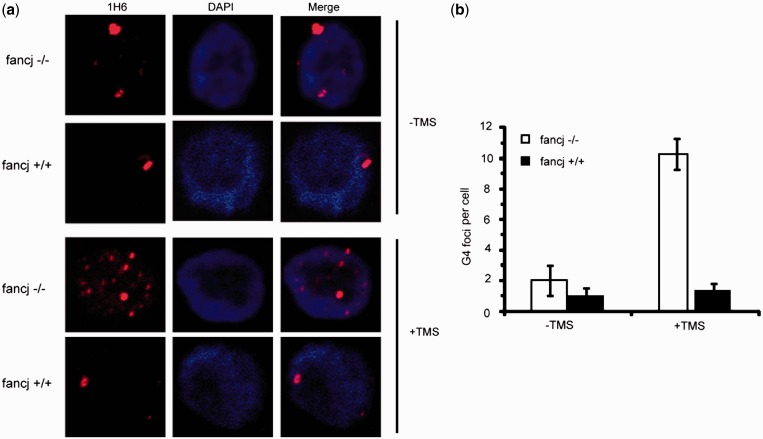
Cells defective in FANCJ show increased 1H6 fluorescence. (**a**) TMS induces elevated numbers of 1H6 foci in FANCJ DT40 chicken cells. FANCJ DT40 cells or cells expressing human FANCJ-WT were either exposed to 5 μM TMS for 2 h or left unexposed, fixed and stained with 1H6 (red) and DAPI (blue). (**b**) Quantification of the change in 1H6 foci with and without TMS treatment. The graph illustrates the mean ± s.e.m of the G4 foci in each cell. More than 100 cells were scored for each sample.

Previously, we observed that depletion of the G4 unwinding helicase FANCJ elevates DNA damage induced by TMS (42). To determine if the effect of TMS is attributable to the accumulation of nuclear G-quadruplex structures in cells deficient for FANCJ, we examined 1H6 staining in a chicken FANCJ knockout cell line transfected with an empty vector or the same plasmid expressing wild-type human FANCJ (Figure 5A). The 1H6 foci were elevated 5-fold in TMS-treated FANCJ/vector cells compared with isogenic FANCJ/FANCJ-WT cells that did not show an increase (Figure 5B), indicating the accumulation of G-quadruplexes in cells lacking FANCJ protein. Untreated cells with or without FANCJ did not show a significant increase in 1H6 foci, indicating that the FANCJ protein may help to unwind TMS-stabilized G4 structures. These results suggest that FANCJ is required to prevent G-quadruplex accumulation in vertebrate cells exposed to TMS. Interestingly, the mean number of 1H6 foci detected in these chicken cells was markedly decreased as compared with human and murine cell lines. It is not known whether this reflects differences in the accessibility or abundance of putative G4-forming sequences in the chicken genome. Nevertheless, the ability to detect enrichment of G4 DNA in FANCJ-deficient cells using the G4-specific antibody 1H6 represents a significant advance in the field that will be useful to determine other genetic or environmental conditions that favor G-quadruplex accumulation. The direct detection of accumulated G-quadruplexes in FANCJ-deficient cells is consistent with recent findings suggesting a prominent role of FANCJ helicase in maintaining epigenetic stability near G4 DNA motifs (43) and the specialization of FANCJ among iron–sulfur cluster DNA helicases to prevent G4-induced damage (44).

Before this study, many different G4 DNA isomers have been assembled *in vitro*, but there was limited direct *in vivo* evidence of their existence in mammalian cells (31,45). Our data are in agreement with a recent support using a phage antibody (31) and support the existence of G4 DNA in mammalian cells. Our data furthermore indicate that the abundance of G4 DNA structures can be modulated by enzymes and specific drugs. The G4-specific monoclonal antibodies described here will be invaluable tools in further studies aimed at understanding the biological role(s) and importance of G4 DNA and its metabolizing enzymes (e.g. helicases) in DNA transactions and genomic stability.

## SUPPLEMENTARY DATA

Supplementary Data are available at NAR Online, including [46–59].

## FUNDING

We acknowledge support from the Canadian Institute of Health Research [MOP38075 and GMH79042 to P.L.] and the Terry Fox Foundation (Program Project Award 018006), the National Institute of Health [GMH79042] and by the Intramural Research program of the NIH, National Institute on Aging; An ERC Advanced grant (to P.L.). Funding for open access charge: BC Cancer Foundation.

*Conflict of interest statement*. None declared.

## Supplementary Material

Supplementary Data
