# Supplementary material for: Detection of G-quadruplex DNA in mammalian cells
Source: Nucleic Acids Res. 2013 Oct 24;42(2):860–9. doi: 10.1093/nar/gkt957 (PMC3902944; doi:10.1093/nar/gkt957)
Supplement: Supplementary Data [file supp_42_2_860__index.html]

Detection of G-quadruplex DNA in mammalian cells — Supplementary Data 

# Detection of G-quadruplex DNA in mammalian cells

## Supplementary Data

files

**Files in this Data Supplement:**

- Supplementary Data - pdf file
- Supplementary Data - pdf file
