# Supplementary material for: Detection of G-quadruplex DNA in mammalian cells
Source: Nucleic Acids Res. 2013 Oct 24;42(2):860–9. doi: 10.1093/nar/gkt957 (PMC3902944; doi:10.1093/nar/gkt957)
Supplement: Supplementary Data [file supp_gkt957_nar-02552-f-2013-File007.pdf]

SUPPLEMENTARY TABLES HENDERSON ET AL.,

Sequence 5' to 3'	G4 DNA Structure	Cation	Name	Reference
d [TGGGGG(TTAGGG)$_2$T]	Tetramer	1 M NaCl	Ver-3	(12)
d (TTTTGGGG)$_2$	Tetramer	1 M NaCl	Oxy-2	(9)
d (CGCGCGCGTTTCGCGCGCG)	GC hairpin	none	GC Hair	(55)
d (TTTTGGGG)$_4$	Basket	100 mM NaCl	Oxy-4	(56)
d (TTGGGG)$_4$	Mixture	100 mM NaCl	Tet-4	(56)
d [AGGG(TTAGGG)$_3$]	Basket	100 mM NaCl	Ver-4	(11)
d (TTAGGGGGTTA)	Tetramer	1 M NaCl	G5 G4	(56)
d (GGTTGGTGTGGTTGG)	Monomeric chair	100 mM KCl	M.Chair	(57)
d [AGGG(TTAGGG)$_3$]	Monomeric basket	100 mM NaCl	M.Bask	(11)
d [AGGG(TTAGGG)$_3$]	Propeller	150 mM KCl	Prop	(58)
d (GGGGTTTTGGGG)	Dimeric basket	100 mM NaCl	D.Bask	(10)
r (UGGGGU)	RNA tetramer	1 M NaCl	RNA G4	(59)
d (TTTCTTTTTTCTTCTTTTCTTTCTTTTTCT)	Triplex	100 mM NaCl	TC30W	(47)
d (AGAAAAAGAAAGAAAAGAAGAAAAAAGAAA)	Triplex	100 mM NaCl	TC30C	(47)
d (TCTTTTTCTTTCTTTTCTTCTTTTTTCTTT)	Triplex	100 mM NaCl	C30	(47)

Table S1. Oligonucleotides used in this study to synthesize DNA or RNA G4 structures. Sequences used for generating DNA or RNA secondary structures. The cation concentration used to generate specific structures is listed; following purification structures were stored in PBS and maintained at -20 °C until use. Assigned G4 molecular structure is derived from native PAGE, CD experiments and referenced material. Soluble competitors included: *Oxytricha* intermolecular G4 (Oxy-2), vertebrate intermolecular G4 (Ver-3), poly d(G), previously designed GC hairpin (55), G5 intermolecular parallel stranded quadruplex (G5 G4), *Oxytricha* intramolecular G4 (Oxy-4), vertebrate intramolecular G4 (Ver-4), *Oxytricha* unfolded monomer, *Oxytricha* duplex and biotin.

Heavy Chain

	CDRH-1							
	**27**			**30**	**35**			**38**
**8H2**	G	Y	T	F	T	S	Y	G
**4E11**	G	Y	T	F	T	D	Y	Y
**5E11**	G	F	A	F	S	S	F	G
**5C10**	G	Y	T	F	T	S	Y	W
**1H6**	G	F	T	F	R	N	Y	W
***mev*IIB4**	G	F	T	F	S	S	F	G

	CDRH-2									
	**56**			**59**			**62**			**65**
**8H2**	I	Y	P	R			S	G	N	T
**4E11**	I	F	P	G			S	G	S	T
**5E11**	I	T	S	G			G	T	Y	T
**5C10**	I	D	P	N			S	G	G	T
**1H6**	I	R	L	K	S	D	N	Y	A	T
***mev*IIB4**	I	S	S	G			S	S	T	L

	CDRH-3														
	**105**														
**8H2**	A	R	V	R	G	G	Y	Y	G	S	S	S	H	W	Y
**4E11**	A	R	G	G	E	L	W	S	S	Y	Y	A	M	D	Y
**5E11**	A	R	H	W	A	Y	Y	S	N	Y	L	F	A	Y	
**5C10**	A	R	S	P	E	I	Y	Y	P	A	W	F	A	Y	
**1H6**	T	N	W	Y	Y	F	D	Y							
***mev*IIB4**	T	S	V	T	V	S	S	A	K	T	T	P			

Light Chain

	CDRL-1										
	**27**					**32**	**34**				**38**
**8H2**	E	S	V	D	N		Y	G	I	S	F
**4E11**	S	S	V	S					S	S	H
**5E11**	Q	S	L	V	H	S	N	G	N	T	Y
**5C10**	K	R	I						S	K	Y
**1H6**	Q	S	L	L	Y	S	N	G	K	T	Y
***mev*IIB4**	K	S	V	S	T		S	G	Y	S	Y

	CDRL-2		
	**56**		**65**
**8H2**	A	A	S
**4E11**	S	T	S
**5E11**	K	V	S
**5C10**	S	G	S
**1H6**	L	V	S
***mev*IIB4**	L	V	S

	CDRL-3									
	**105**									
**8H2**	Q	Q	S	K	E	V	P	A	R	T
**4E11**	Q	Q	Y	S	G	Y	P	L	N	T
**5E11**	S	Q	S	T	H	V	P	P	T	
**5C10**	Q	Q	H	N	E	F	P	L	T	
**1H6**	V	Q	G	T	H	F	P	L	T	
***mev*IIB4**	Q	H	I	R	E	L	Y	T		

Table S2. Variable heavy and light chain amino acid composition of purified monoclonal antibodies. Amino acid composition of CDRs for purified monoclonal antibodies illustrates variability of amino acids. CDRs contain the contact points between antibodies and target ligands. Light grey shading highlights conserved amino acids in at minimum four antibodies. Red highlighted amino acids (basic residues) have been previously shown to directly interact with nucleic acid molecules.

REFERENCES

9. Sen, D. and Gilbert, W. (1990) A sodium-potassium switch in the formation of four-stranded G4-DNA. Nature, 344, 410-414.
10. Smith, F.W. and Feigon, J. (1992) Quadruplex structure of Oxytricha telomeric DNA oligonucleotides. Nature, 356, 164-168.
11. Wang, Y. and Patel, D.J. (1993) Solution structure of a parallel-stranded G-quadruplex DNA. Journal of molecular biology, 234, 1171-1183.
12. Williamson, J.R. (1994) G-quartet structures in telomeric DNA. Annual review of biophysics and biomolecular structure, 23, 703-730.
47. Wu, Y., Rawtani, N., Thazhathveetil, A.K., Kenny, M.K., Seidman, M.M. and Brosh, R.M., Jr. (2008) Human replication protein A melts a DNA triple helix structure in a potent and specific manner. Biochemistry, 47, 5068-5077.
55. Carrasco, C., Rosu, F., Gabelica, V., Houssier, C., De Pauw, E., Garbay-Jaureguiberry, C., Roques, B., Wilson, W.D., Chaires, J.B., Waring, M.J. et al. (2002) Tight binding of the antitumor drug ditercalinium to quadruplex DNA. Chembiochem : a European journal of chemical biology, 3, 1235-1241.
56. Williamson, J.R., Raghuraman, M.K. and Cech, T.R. (1989) Monovalent cation-induced structure of telomeric DNA: the G-quartet model. Cell, 59, 871-880.
57. Macaya, R.F., Schultze, P., Smith, F.W., Roe, J.A. and Feigon, J. (1993) Thrombin-binding DNA aptamer forms a unimolecular quadruplex structure in solution. Proceedings of the National Academy of Sciences of the United States of America, 90, 3745-3749.
58. Parkinson, G.N., Lee, M.P. and Neidle, S. (2002) Crystal structure of parallel quadruplexes from human telomeric DNA. Nature, 417, 876-880.
59. Cheong, C. and Moore, P.B. (1992) Solution structure of an unusually stable RNA tetraplex containing G- and U-quartet structures. Biochemistry, 31, 8406-8414.
