# Supplementary material for: Detection of G-quadruplex DNA in mammalian cells
Source: Nucleic Acids Res. 2013 Oct 24;42(2):860–9. doi: 10.1093/nar/gkt957 (PMC3902944; doi:10.1093/nar/gkt957)
Supplement: Supplementary Data [file supp_gkt957_nar-02552-f-2013-File008.pdf]

SUPPLEMENTARY DATA

G-Quadruplex Oligonucleotides

Oligonucleotides were synthesized using an ABI 394 DNA/RNA synthesizer by the University of Calgary DNA Synthesis lab. Both 5'-amino and 3'-biotin modifications were incorporated into DNA oligonucleotides at the time of synthesis. A list of oligonucleotides used in this study is depicted in **Supplementary Table 1**. For unimolecular and bimolecular G4 structures, oligonucleotides were resuspended at a final concentration of 0.1 mM in 10 mM Tris-HCl, 1 mM EDTA (TE). Solutions were heat-denatured for 4 min at 95°C before the addition of 100-150 mM (final) NaCl or KCl. Samples were allowed to slowly equilibrate to room temperature over 5-8 hr. For tetramolecular G4 structures, oligonucleotides were resuspended at a final concentration of 1 mM in TE. Samples were heat denatured at 100°C for 2 min and subsequently placed on ice prior to the addition of 1 M (final) NaCl. Cooled samples were sealed in microcentrifuge tubes and incubated at 50°C for 72 hr. Duplex DNA and the previously designed GC hairpin (46) were generated by heat denaturation at 95°C for 2 min and annealing complementary sequences at room temperature. Single stranded oligonucleotides were maintained in TE and underwent 2 min heat denaturation at 95 °C prior to use. The previously designed triplex structure (47) was generated by first annealing the complementary sequences (TC30W + TC30C) into a duplex structure. To prepare a stable triplex, the inverse 30-mer strand was annealed to the 30 bp duplex in a 1:2 molar ratio in the presence of 33 mM Tris-acetate (pH 5.5) and100 mM NaCl. Oligonucleotide complexes were separated from unbound oligonucleotides by native polyacrylamide electrophoresis and gel purification.

Immunization

Reactive sulfhydryl groups were generated on key holelimpet hemocyanin (KLH) (Sigma) by the addition of a 20-fold molar excess of Traut’s reagent (Pierce). The heterobifunctional crosslinker Sulfo-MBS (Pierce) was used to conjugate the amino modified G4 DNA and reactive KLH to produce KLH-G4 conjugates. KLH-G4 conjugates were emulsified (1:1) in Complete Freund’s Adjuvant (CFA) and used in immunizations of C57BL/6J mice obtained from the Jackson Laboratories (Bar Harbor, Maine). Groups of four 6-8 week old mice (Jackson Laboratories) were immunized 4-5 times at 2 week intervals with 50 µg of KLH-G4 DNA emulsified in CFA (Sigma) for primary immunization or in IFA (Sigma) for the subsequent immunizations, via intraperitoneal injection.

Serum Screening and Hybridoma Generation

For screening of anti-G4 DNA antibodies, streptavidin-coated (1.5 µg/mL) 96-well plates (Falcon) were blocked for 1 hr at 22ºC with 2% Bovine Serum Albumin (BSA) in Phosphate-buffered Saline (PBS) containing 0.1% Tween-20. After 3 washes with PBS/0.1% Tween-20 (PBST), the plates were incubated at 22ºC for 1 hr with 1 µg/35 µl of biotinylated-G4 DNA diluted in PBS. After 3 washes with PBST, serial dilutions of mouse sera diluted in PBS with 1% BSA were incubated at 22ºC for 2 hr. Upon 6 washes with PBST, bound anti-DNA antibodies were detected with HRP labeled goat-anti-mouse IgG (Sigma) and identified using ABTS (Sigma) as a substrate. Antibody binding was quantified by spectrophotometry at 405 nm. Splenocyte/myeloma fusions were performed for highest serum responders according to the method described by Köhler and Milstein (48). Hybridoma supernatants were screened by standard ELISA assays using 96-well plates coated with 1 µg/35 µl biotinylated-G-quadruplex DNA bound to streptavidin (Sigma).

Single-cell Cloning and Antibody Purification

Upon positive identification by ELISA, cells were harvested and transferred to 1.5 cm Petri dish plates containing semi-solid medium: 1.5 ml of IMDM supplemented with 0.9% methylcellulose (StemCell Technologies), 10% FCS, 2 mM L-glutamine (Sigma) and 75 units of IL-6. Clones were picked from methylcellulose plates and transferred to individual wells of a 96-well plate in liquid media supplemented with 10% fetal calf serum and 50 units of IL-6. Clones were cultured for four days and supernatants from selected clones were screened by ELISA. Three rounds of single cell cloning were performed before hybridomas were considered monoclonal. Antibodies were purified from hybridoma supernatants by affinity chromatography using HiTrap protein G HP columns (GE Healthcare) on an ÄKTA Purifier instrument (Amersham Biosciences) according to manufacturer's protocol. Purified antibodies were then buffer-exchanged into PBS by a Fast Desalting column HR10/10 (Pharmacia).

Isotype, Affinity, and CDR Sequencing

Isotyping was performed by ELISA and quantified by spectrophotometry following manufacturer's instructions (Pierce). Apparent antibody affinity was determined by binding curve analysis (49,50). Biotinylated versions of immunizing G4 DNA was captured on streptavidin coated ELISA plates and probed with various concentrations of antibodies to generate a binding curve for each antibody G4 combination. Apparent affinities were determined from the resulting binding curves by nonlinear regression and the antibody concentration at half-maximal binding using GraphPad Prism software. Variable heavy and light chains of each hybridoma were sequenced following Wang *et al.* (51). Purified amplicons were sequenced by the NAPS unit (Nucleic Acid Protein Service Unit, University of British Columbia) using M13 forward and reverse primers and analyzed using IMGT/V-Quest program (International ImMunoGeneTics Information System). Alignment of the primary amino acid

sequences was performed using IMGT/V-Quest program (http://imgt.cines.fr/,) and compared to an originally purified and sequenced antibody from Brown *et al*. (28).

Cell Lines

Cell lines used in this study included: primary human foreskin fibroblast cell line BJ (ATCC #CRL-2522), cervical adenocarcinoma cell line HeLa (ATCC# CCL-2), ALT cell line GM847, U2OS (ATCC #HTB-96), chicken DT40 cells and normal mouse embryonic stem cells (52). All cell lines were maintained in a humidified chamber at 37^{0}C and 5% CO_2. Cells were cultured in tissue culture treated flasks and maintained in standard growth medium. Generation of cell lines with fancj knockout and complemented with human FANCJ-WT have been described elsewhere (53,54).

Immunohistochemistry

Normal human tissues were obtained from the Centre for Translational and Applied Genomics (CTAG) at the BC Cancer Agency. Tissues were fixed, embedded in paraffin, sectioned and stained on site at CTAG using a Tissue-Tek Xpress continuous rapid tissue processor and the Discovery XT for detection. Images were obtained using a Zeiss Axioplan microscope and OpenLab software (Improvision).

In situ hybridization

Prior to harvesting cells used for *in situ* hybridization, 1:1000 dilution of colecemid was added to cells for 3 hrs at 37 ˚C. Cells were then resuspended in 75 mM KCl (Hypo) for 10 minutes at 37 ˚C. Cells were subsequently fixed in fresh methanol:acetic acid solution. Slides are then digested with pepsin at 1 µg/µl in 0.01N HCl (pH 2.0) for 10 mins at 37 ˚C. Metaphases were then subjected to RNase A treatment at 0.5 µg/ml for 10 mins at 37 ˚C. Primary antibodies were subsequently bound to metaphases following methods above, followed by the addition of peptide nucleic acid (PNA) probes $(CCCTAA)_3$ labeled with Cy3, as previously reported (52). (Images were acquired with the Deltavision RT (Applied Precision, Issaquah, Washington) microscope using a 60x plan apochromatic 1.4 NA oil objective (Olympus). For each cell, typical stacks of 760x760 pixels x 25 images were acquired. Exposure was 0.15-0.3 seconds and neutral density filters (transmission 32%) were used to minimize photobleaching effects. Three-dimentional reconstruction and deconvolution were performed using Softworx Suite (Applied Precision).

Flow Cytometry

Where indicated, cells were incubated with varying concentrations of TMPyP4 for 24hr. Cells were trypsinized, fixed in 4% paraformaldehyde (Sigma), and then permeabilized with 0.5% Tween 20 in

PBS. Cells were then blocked in 5% normal goat serum for 4 hr at room temperature. Subsequently, suspended cells were incubated with 8 μg/ml (final concentration) 1H6 antibody for 12 hr at 4ºC. After washing cells were resuspended in PBS containing 1:500 dilution of secondary antibody conjugated with PE for 1 hr at 25ºC. Stained cells were analyzed using a FACSCalibur (BD Biosciences). Data were recorded and analyzed using CellQuest (BD Biosciences) and FlowJo (Tree Star, Inc.) in succession.

SUPPLEMENTARY FIGURE LEGENDS

A

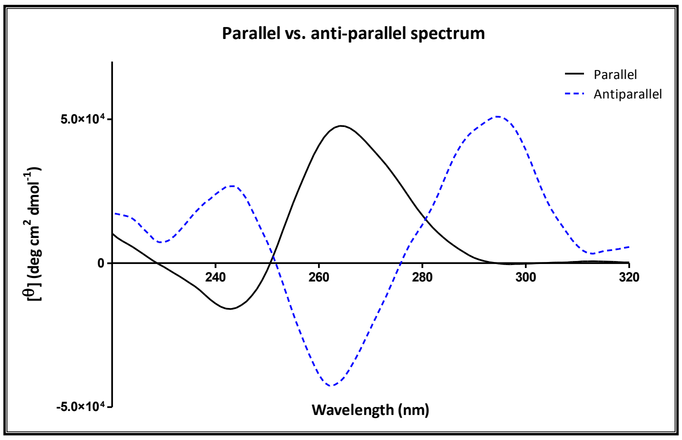

B

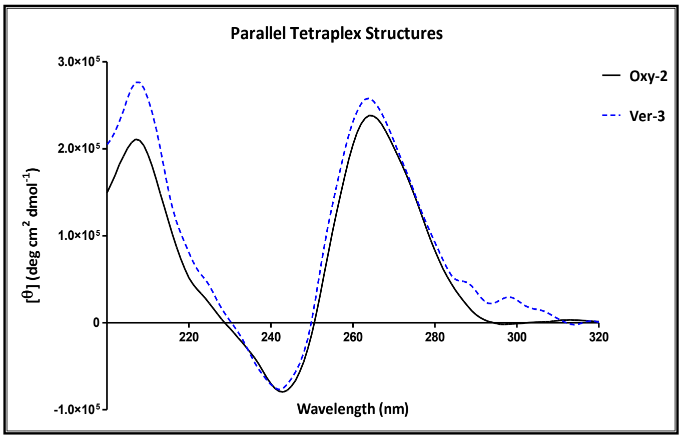

Figure S1. CD spectrum of parallel G4 DNA have different ellipticity maximum and minimum than antiparallel G4 DNA structures. (A) An example of the differences between characteristic parallel G4 DNA and antiparallel G4 DNA. Characteristic parallel CD spectrum contains a molar ellipticity maximum at ~260 nm and a minimum at ~240 (Purified tetramolecular Ver-3 is shown as classical parallel example). While, characteristic antiparallel CD spectrum contain a molar ellipticity maximum at ~290 nm and a minimum at ~260 nm (Purified dimeric basket is shown as classical antiparallel example. Classical CD spectrum of tetramolecular G4 DNA. (B) Circular dichroism spectroscopy of immunizing tetramolecular G4 DNA structures show characteristic tetraplex ellipticity maximum around 260 nm and minimum around 240 nm. Telomeric oligonucleotide Oxy-2 and Ver-3 were incubated in appropriate conditions (See Methods) for the formation of parallel-stranded tetramolecular G-quadruplex DNA.

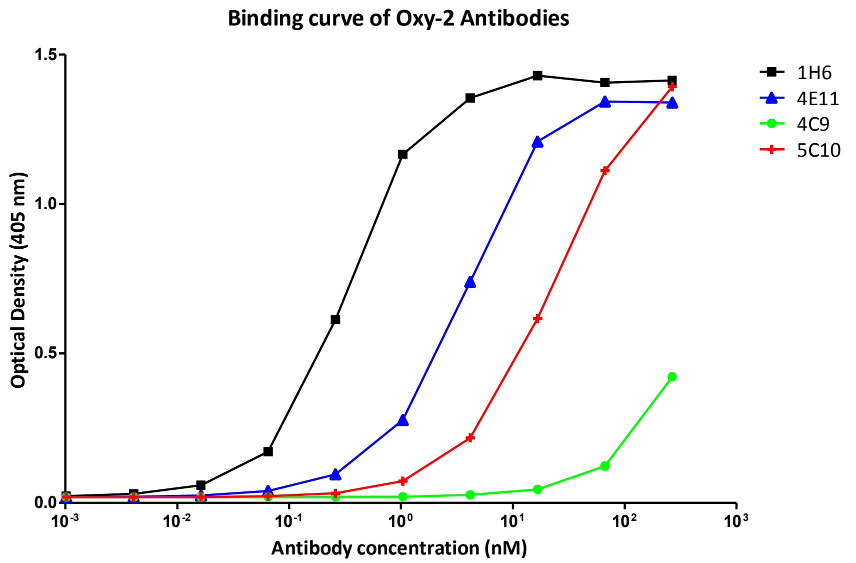

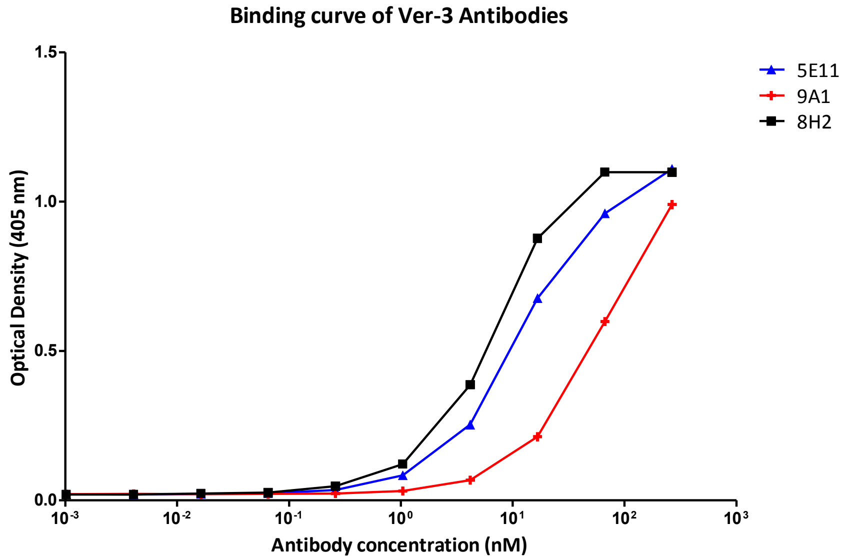

Figure S2. Binding curve analysis of antibodies to different G4 DNA immunogens. Biotinylated G4 DNA structures were coated on ELISA plates and used to screen purified monoclonal antibodies. Serially diluted antibodies give rise to binding curves for the determination of apparent affinity. Legends indicate the antibody name and corresponding color-coded binding curve. Sequences used for G4 generation and subsequent binding analysis include: Ver-3 d[TGGGGG(TTAGGG)$_2$T], Oxy-2 d[TTTTGGGG]$_2$ and Tet-4 d[TTGGGG]$_4$.

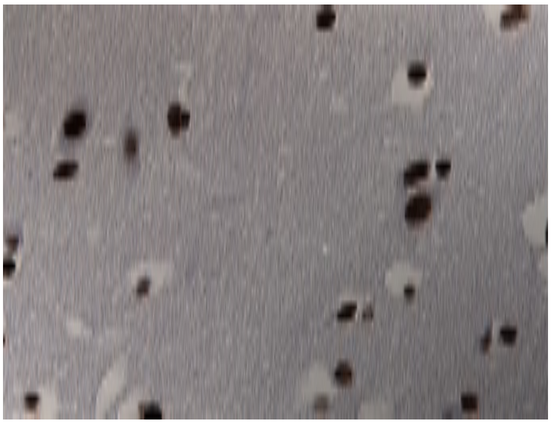

Brain

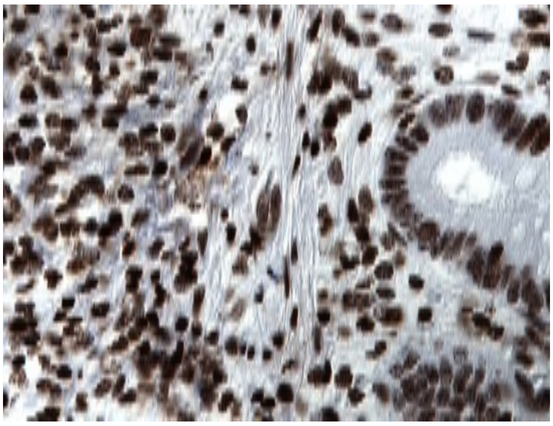

Appendix

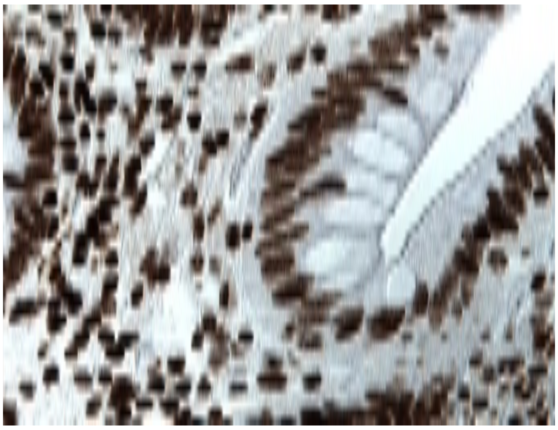

Colon

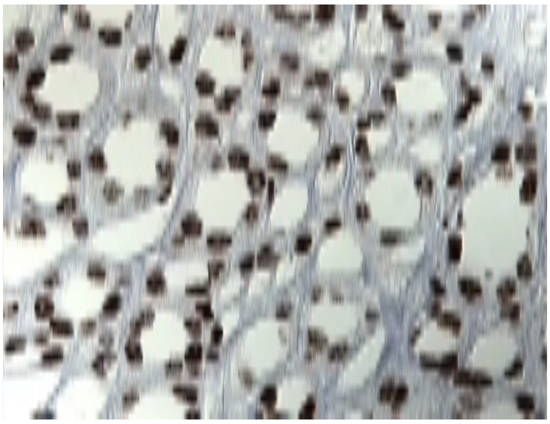

Kidney

Figure S3. Normal human tissue sections were used for immunohistochemical staining with 1H6 antibodies (dark brown). Representative images from the brain, appendix, colon and kidney.

A

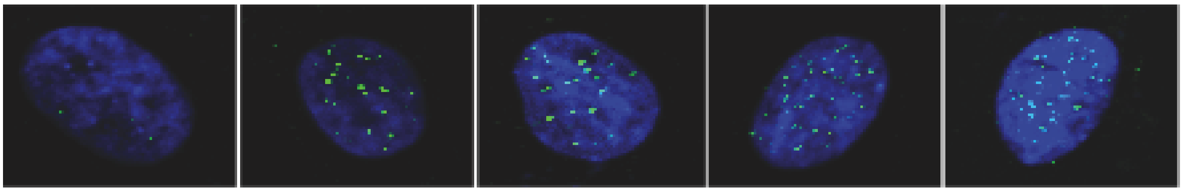

Time: 0 mins 30 mins 60 mins 3 hours 8 hour

B

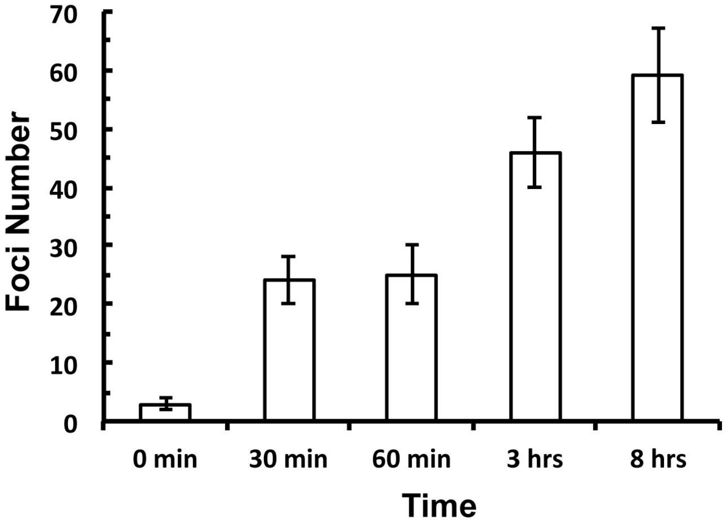

***Henderson et al*.**

Figure S4. **(A)** Fluorescence microscopy analysis of fixed cells after time course experiment of 5 μM telomestatin shows increased number of antibody foci when stained with 1H6 antibody. U2OS cells were treated with telomestatin (TMS) for the indicated amount of time prior to fixation and staining with antibody 1H6**.** **(B)** Quantification of 1H6 foci upon time course treatment of U2OS cells with telomestatin. 100 cells were examined for each cell line/treatment. Error bars represent s.e.m.

SUPPLEMENTAL DATA REFERENCES

46. Giraldo, R. and Rhodes, D. (1994) The yeast telomere-binding protein RAP1 binds to and promotes the formation of DNA quadruplexes in telomeric DNA. The EMBO journal, 13, 2411-2420.
47. Wu, Y., Rawtani, N., Thazhathveetil, A.K., Kenny, M.K., Seidman, M.M. and Brosh, R.M., Jr. (2008) Human replication protein A melts a DNA triple helix structure in a potent and specific manner. Biochemistry, 47, 5068-5077.
48. Kohler, G. and Milstein, C. (1975) Continuous cultures of fused cells secreting antibody of predefined specificity. Nature, 256, 495-497.
49. Pantophlet, R., Ollmann Saphire, E., Poignard, P., Parren, P.W., Wilson, I.A. and Burton, D.R. (2003) Fine mapping of the interaction of neutralizing and nonneutralizing monoclonal antibodies with the CD4 binding site of human immunodeficiency virus type 1 gp120. J Virol, 77, 642-658.
50. Lin, C.W. and Wu, S.C. (2003) A functional epitope determinant on domain III of the Japanese encephalitis virus envelope protein interacted with neutralizing-antibody combining sites. J Virol, 77, 2600-2606.
51. Wang, Z., Raifu, M., Howard, M., Smith, L., Hansen, D., Goldsby, R. and Ratner, D. (2000) Universal PCR amplification of mouse immunoglobulin gene variable regions: the design of degenerate primers and an assessment of the effect of DNA polymerase 3' to 5' exonuclease activity. J Immunol Methods, 233, 167-177.
52. Ding, H., Schertzer, M., Wu, X., Gertsenstein, M., Selig, S., Kammori, M., Pourvali, R., Poon, S., Vulto, I., Chavez, E. et al. (2004) Regulation of murine telomere length by Rtel: an essential gene encoding a helicase-like protein. Cell, 117, 873-886.
53. Wu, Y., Sommers, J.A., Suhasini, A.N., Leonard, T., Deakyne, J.S., Mazin, A.V., Shin-Ya, K., Kitao, H. and Brosh, R.M., Jr. (2010) Fanconi anemia group J mutation abolishes its DNA repair function by uncoupling DNA translocation from helicase activity or disruption of protein-DNA complexes. Blood, 116, 3780-3791.
54. Kitao, H., Nanda, I., Sugino, R.P., Kinomura, A., Yamazoe, M., Arakawa, H., Schmid, M., Innan, H., Hiom, K. and Takata, M. (2011) FancJ/Brip1 helicase protects against genomic losses and gains in vertebrate cells. Genes Cells, 16, 714-727.
